# Physiological condition of nestling great tits *Parus major* in response to experimental reduction in nest micro- and macro-parasites

**DOI:** 10.1093/conphys/coy062

**Published:** 2018-11-22

**Authors:** Michał Glądalski, Adam Kaliński, Jarosław Wawrzyniak, Mirosława Bańbura, Marcin Markowski, Joanna Skwarska, Jerzy Bańbura

**Affiliations:** 1 Department of Experimental Zoology and Evolutionary Biology, Faculty of Biology and Environmental Protection, University of Łódź, Banacha 12/16, 90-237 Łódź, Poland; 2 Museum of Natural History, Faculty of Biology and Environmental Protection, University of Łódź, Kilińskiego 101, 90-011 Łódź, Poland

**Keywords:** Body condition, environmental stress, haematology, nest, *Parus major*, physiological condition, urban populations, wild populations

## Abstract

Most passerines use nests as the exclusive place to lay and incubate eggs and bring nestlings up to fledging. Nests of secondary cavity nesters, like tits, provide a moist, warm and protected habitat for reproduction of blood parasites. Offspring fitness depends on interactions between parental care and environmental constraints. Life-history theory suggests that macro- and micro-parasites may generate selection pressures by affecting host health. In the present study, we replaced natural great tit *Parus major* nests in two, structurally and floristically contrasting sites (an urban parkland and a rich deciduous forest, located 10 km apart in Łódź, central Poland), with fresh, sterilized, artificial moss-cotton wool nests, twice, on the fifth and tenth day of nestlings life. We then examined haematological condition indicators (haemoglobin and glucose concentrations) of about 14-day-old nestlings. Nestlings that were developing in treated nests improved their health status in comparison with control nestlings. The mean haemoglobin and glucose concentrations (treated and control) also varied between both study areas. Our study confirms that the level of haemoglobin and especially the level of glucose may be treated as reliable indicator of environmental characteristics in great tits.

## Teaser

Life-history theory suggests that macro- and micro-parasites may generate selection pressures by affecting host health. In the present study, we replaced natural great tit *Parus major* nests in two, structurally and floristically contrasting sites. Nestlings that were developing in treated nests improved physiological health status in comparison with control nestlings.

## Introduction

Avian nests are multi-functional structures which provide a safe environment for parents, eggs and nestlings (Collias and Collias, 1984; Heenan, 2013; Mainwaring *et al.*, 2014). They provide thermal insulation, help to maintain microclimate, protect from predators, help to maintain position of eggs during incubation, thus supporting general nestling body condition and survival (Deeming, 2011; Álvarez *et al.*, 2013; Heenan, 2013; Glądalski *et al.*, 2016a; Lambrechts *et al.*, 2014, 2017; Maziarz *et al.*, 2017; Wesołowski and Wierzcholska, 2018). But avian nests accommodate more than just birds and their offspring. Nests offer also a moist, warm and protected environment with regular food supply and therefore convenient breeding habitats for a large diversity of invertebrate ectoparasites and micro-organisms. Furthermore, many of those invertebrate ectoparasites are vectors of pathogens—viruses, bacteria, fungi and protozoa (Heylen and Matthysen, 2010, 2011; López-Rull and Macías Garcia, 2015). Additionally, secondary hole nesters, like tits *Paridae*, often use natural holes (and nest boxes) not only to nest, but also to spend nights during non-breeding periods, or they visit their neighbours’ nest boxes which may induce parasite and pathogen transmission among different nest sites (Gosler, 1993; Stenning, 2018). Therefore, birds may be host to a variety of parasites (mostly flying and non-flying insects and arachnids), including fleas, flies, lice, mites, leaches, ticks or fungi. Those ectoparasites are harmful to their hosts by draining resources and cause damage that can be subtle, severe or even lethal and have been shown to influence individual survival and fitness. Life-history theory assumes that an individual cannot invest equal amounts of resources into all its needs, and allocation of limited resources to different functions of an organism causes numerous tradeoffs between competing needs (Price, 1980; Hurtrez-Boussès *et al.*, 1997, 1998; Heylen *et al.*, 2013). Birds evolved various (behavioural and physiological) anti-parasite strategies (e.g. in mate choice, trough avoidance of infested holes, higher nest sanitation rates in parasitized nests, an increase of feeding visits by parents in infested broods or using aromatic plants as potential repellents), but nestlings themselves are particularly vulnerable to parasites because their physiological and behavioural defenses are not fully developed (Mennerat *et al.*, 2009; López-Rull and Macías Garcia, 2015; Mainwaring, 2017). In general, parasites have the potential to alter the life-history traits of their hosts and may act as a selective pressure forcing their evolution (Hamilton and Zuk, 1982; Poulin, 1995; Thomas *et al.*, 2000). The prevalence and the impact of those parasites on their avian hosts is currently under study and it is crucial to assess and interpret current damage for the understanding of the co-evolution of host-parasite interactions (Heylen and Mattyhysen, 2011; Reynolds *et al.*, 2016; Hanmer *et al.*, 2017; Mainwaring, 2016, 2017).

Haematological parameters are among the most direct indicators of physiological condition and are recently widely used in field and experimental studies of birds and mammals (Norte *et al.*, 2008; Johnstone *et al.*, 2015; Podlaszczuk *et al.* 2017; Kaliński *et al.* 2016, 2017a). The concentration of haemoglobin is positively related to the physiological condition of nestling altricial birds, which depend on the quantity and quality of food delivered to them by parents (Lill *et al.*, 2013; Minias, 2015; Kaliński *et al.*, 2017b). Haemoglobin in the blood carries oxygen from the respiratory organs to the tissues where it releases the oxygen and provides energy to power the functions of the organism. In general, changes in the level of haemoglobin could be caused by such factors as nutritional deficiency, hydration, energy expenditure, parasite pressure, age, sex or genetics (Stevens, 1996; Lill *et al.*, 2013). Long-term studies on blue tits *Cyanistes caeruleus* and great tits *Parus major* at our study areas in central Poland suggest that a higher level of haemoglobin is positively related to good physiological condition of nestlings (Kaliński *et al.*, 2015a; Glądalski *et al.*, 2016b).

Glucose concentration is considered a reverse and less robust indicator of physiological condition than haemoglobin, but is also useful in ecophysiology of birds (Lill, 2011; Minias and Kaczmarek, 2013; Minias, 2014; Glądalski *et al.*, 2015). In general, glucose concentration mainly reflects metabolic rates of animals (Brown, 1996). Long-term studies of blue tit and great tit populations at our study areas suggest that a higher level of glucose is negatively related to the physiological condition of nestlings (Kaliński *et al.*, 2014, 2015b).

A negative relation between the concentration of haemoglobin and glucose would be expected, as recently reported by Minias (2014) and Glądalski *et al.* (2015).

However, some recent studies suggest caution when using blood characteristics to evaluate body condition and it is advised not to automatically extend results for a particular population to other populations or species (Fair *et al.*, 2007; Krams *et al.*, 2010; Prinzinger and Misovic, 2010; Lill *et al.*, 2013). Additionally, Krams *et al.* (2013) states that studies on the effects of parasites are still in their infancy, awaiting more experimental research both in wild and captive birds. Currently, relatively few landscape ecologists employ physiological approaches to evaluate habitat quality. The development of conservation physiology may help with identifying early warning signs of populations in trouble and may play an important role in monitoring and assessing the progress of habitat restoration effects (Cook *et al.*, 2013; Cooke *et al.*, 2017).

We showed that great tits in our forest study area produce larger clutches and more fledglings than birds in the parkland study area (Wawrzyniak *et al.*, 2015). A large part of this variation is probably related to the observed difference in the abundance of insect food between our study areas. The study areas significantly differed in their insect productivity, including caterpillar productivity, with caterpillars being 2–5 times more abundant in the forest site than in the parkland site (Marciniak *et al.*, 2007; Wawrzyniak *et al.*, 2015). Our previous (Słomczyński *et al.*, 2006) and unpublished studies suggest that similar numbers of species and amounts of parasites occur in nests from both our study areas. It could suggest that the experimental treatment should improve health status similarly.

In this study, we test if the experimental reduction in nest blood feeding ectoparasites and nest-specific micro-organisms may affect body condition and therefore offspring fitness of great tit nestlings. As simple biochemical indicators of nestling condition we use haemoglobin (g/l) and glucose (mg/dl) concentrations in the blood of about 14-day-old nestlings. Our hypothesis is that sterile nests should drastically reduce parasitic and pathogen pressure and therefore similarly improve the health status of nestlings (higher mean level of haemoglobin and lower mean level of glucose, as glucose is considered as inverted index of body condition) in both study areas.

## Material and Methods

### Study sites

This experimental study, carried out in 2018, is part of a long-term project of research on the breeding biology of secondary cavity nesters around Łódź, central Poland (Bańbura *et al.*, 2011; Glądalski *et al.*, 2018). Study areas were located in two structurally and floristically contrasting types of habitats. The urban parkland study site (51°45′N; 19°24′E), ca. 80 ha, has a fragmented tree cover (formed artificially, Glądalski *et al.*, 2016c). The forest study site (51° 50′N, 19° 29′E), ca. 130 ha area in the interior of a mature mixed deciduous forest (1 250 ha in total) with oaks as predominating tree species. During the breeding season, the nest boxes were inspected every 5 days (or more often if needed) to record basic breeding characteristics, including nestling age. Both study sites were supplied with about 500 standard wooden nest boxes (Lambrechts *et al.*, 2010); about 200 in the urban parkland area and about 300 in the forest area (Glądalski *et al.*, 2016c).

### General field and experimental procedures

The experimental treatment took place in 2018. Of 65 complete and incubated clutches of great tits, 23 were blindly drawn to the experiment (11 in the urban parkland study site and 12 in the forest study site) and 42 were control broods (12 in the urban parkland study site and 30 in the forest study site). On the 5th day after hatching, all the natural nests were swapped for the first time with man-made, artificial nests. All the nestlings were placed in the nest cup. On the 10th day, the first artificial nests were swapped for the second time with new artificial nests (this was expected to reduce the number of new parasites that could have infested the nest since the first nest swap). All the nestlings were also placed in the nest cup. We also used a positive control; in control nests all the nestlings were removed twice from the nest (for a similar period of time and in similar stages of nestling development as nestlings from the treated nests).

The artificial nests were constructed of dry moss (the structural nest layer) and cotton wool (100%) for lining of the nest cup. The moss was collected from the local environment. Collected moss (for experimental purposes) was (earlier, before experimental treatment) dried for 48 h in a lab at 25°C. The arthropods present in the collected moss were removed at this stage. During the experimental treatment, bits of moss were used to form and imitate the dimension of the structural section of the natural nest to be swapped, the nest cup was shaped and lined with cotton wool. After those procedures, the artificial nest was put into the nest box. All the nestlings were placed in the artificial nest. Using sterile cotton and moss, we constructed artificial nests with a largely reduced load of pathogens and parasites. We did record no case of the presence of ectoparasites in the artificial nests and we recorded that 100% of natural nests contained at least one kind of parasitic arthropods (lice, fleas, ticks, mites or blowflies). We also assume that we reduced the presence of fungi, bacteria and micro-parasites, normally present in natural nests. In conclusion, the experiment consisted of pathogen/parasite reduced, treated (artificial) nests and natural, control nests (not changed).

### Physiological measurements

When the nestlings of the great tit were 13–14-days-old, they were banded with individually numbered metal rings and measured (wing length, to the nearest 1 mm). A random subsample of three nestlings out of same-age nestlings from every brood were designated for blood sampling (Kaliński *et al.*, 2011). Samples of 5 μl of blood were taken from the ulnar vein of nestlings to HemoCue cuvettes and analyzed in the field using a portable HemoCue Hb 201+ photometer to measure haemoglobin concentration (g/l). In the case of avian blood, this photometer shows haemoglobin values slightly higher than cyanomethaemoglobin spectrophotometry, as described by Eklom and Lill (2006) and Simmons and Lill (2006). A portable HemoCue Glucose 201+ photometer (HemoCue AB, Angelholm, Sweden) was used to analogously establish glucose concentration (mg/dL) in a second sample of blood (also 5 μl). All field procedures were carried out between 9.00 and 14.00 h. During the experimental treatment, 65 broods of the great tit (comprising 556 nestlings) were banded and measured and blood samples from 190 nestlings taken.

### Statistical analyses

Values of haemoglobin concentration and glucose concentration in the blood of nestlings from the same brood were not independent. Therefore, the individual nestling values which were treated as unit records, were analysed using mixed linear models, with brood ID being included as a random factor to control for clustering; degrees of freedom were approximated by the Satterthwaite method (Heck *et al.*, 2010). All the models included wing length as an age-controlling covariate (Crawley, 2002). Experimental treatment/control and sites were treated as fixed factors in these models. Linear mixed modelling was performed using IBM SPSS Statistics 22 software (Heck *et al.*, 2010, 2012; IBM SPSS 22, 2013).

## Results

**Figure 1:**
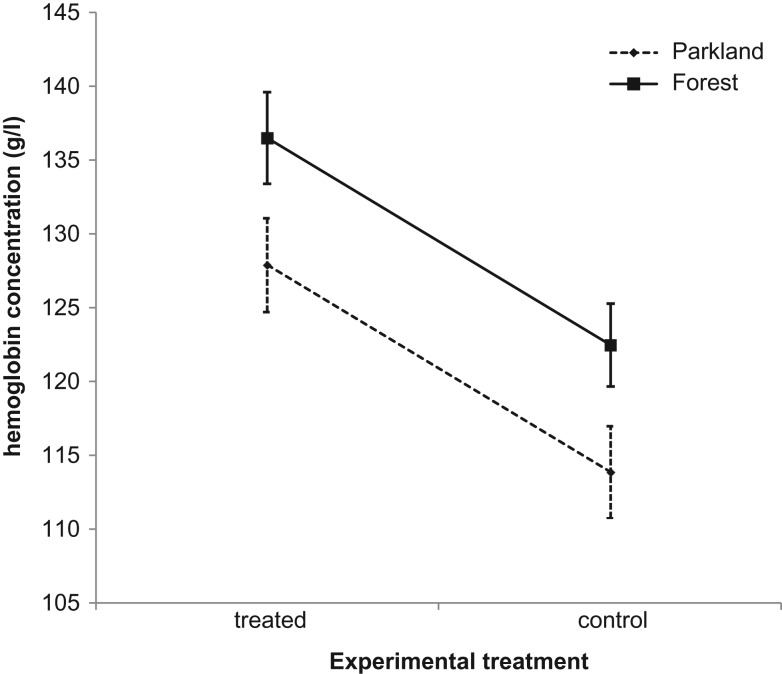
Mean haemoglobin concentrations (g/l) in experimentally treated nests (treated nests with replaced, artificial nests) and control nests in the parkland study area and in the forest study area, with wing length being used as an age-controlling covariate (data shown as mean ± SE).

Minimum value of per-brood mean haemoglobin level in the parkland site was 54.7 g/l (control nest) and 117.3 g/l (treated nests) and maximum per-brood mean was 140.7 g/l (control nest) and 143.0 g/l (treated nests). Minimum value of per-brood mean haemoglobin concentration in the forest site was 100.7 g/l (control nest) and 126.0 g/l (treated nests) and maximum per-brood mean was 139.0 g/l (control nest) and 140.0 g/l (treated nests). The great tit nestlings from artificial nests had on average 14 g/l higher haemoglobin concentration than the control nestlings in both parkland and forest study areas (Table 1, Fig. 1). The mean haemoglobin concentration varied between both study areas and was on average 8.6 g/l higher in the forest study area than in the parkland study area in both treated and control nestlings (Table 1, Fig. 1).

**Table 1:** Summary of a linear mixed model analysis for haemoglobin and glucose concentrations in the blood of great tit nestlings. Effects of study area and experimental treatment are given (mean wing length as covariate, significant values are in bold). Non-significant effects were removed.

Factor (covariate)	Df	*F*	*P*
**Haemoglobin**			
Intercept	1; 182.7	31.8	**<0.001**
Study area	1; 61.5	6.5	**0.013**
Experiment	1; 60.7	17.6	**<0.001**
Wing length (cov)	1; 183.6	11.1	**0.001**
Removed non-significant effects			
Study area * exp. * wing l.	1; 182.0	0.8	0.388
Study area * wing length	1; 179.7	0.0	0.991
Experiment * wing length	1; 184.0	2.0	0.159
Study area * experiment	1; 59.8	1.9	0.171
**Glucose**			
Intercept	1; 124.0	3.2	0.076
Study area	1; 62.1	18.7	**<0.001**
Experiment	1; 60.0	11.3	**0.001**
Wing length (cov)	1; 124.9	9.9	**0.002**
Removed non-significant effects			
Study area * exp. * wing l.	1; 141.6	1.5	0.217
Study area * experiment	1; 58.7	0.0	0.906
Experiment * wing length	1; 144.2	0.1	0.820
Study area * wing length	1; 120.1	0.3	0.596

**Figure 2:**
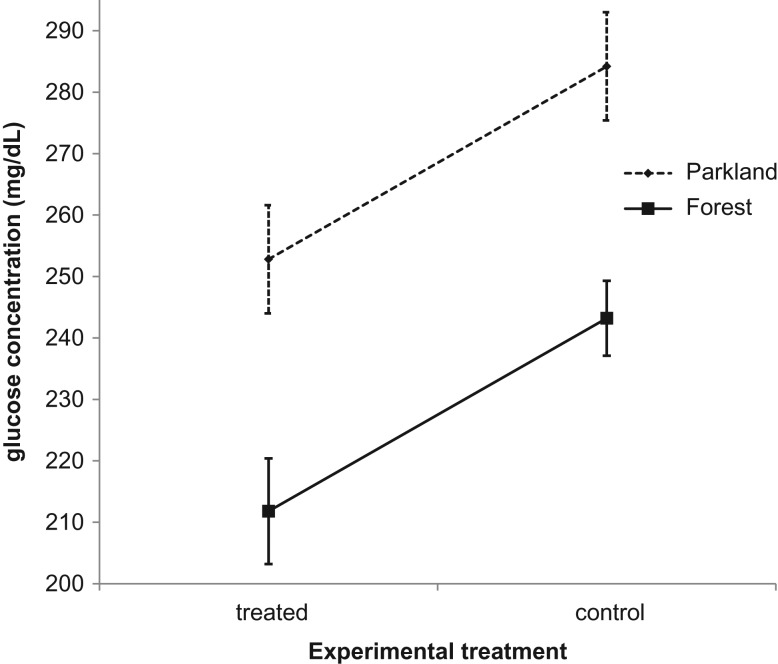
Mean glucose concentrations (mg/dl) in experimentally treated nests (treated nests with replaced, artificial nests) and control nests in the parkland study area and in the forest study area, with wing length being used as an age-controlling covariate (data shown as mean ± SE).

Minimum value of per-brood mean glucose level in the parkland site was 171.7 mg/dl (control nest) and 229.0 mg/dl (treated nests) and maximum per-brood mean was 400.0 mg/dl (control nest) and 298.7 mg/dl (treated nests). Minimum value of per-brood mean glucose concentration in the forest site was 186.0 mg/dl (control nest) and 176.0 mg/dl (treated nests nest) and maximum per-brood mean was 333.0 mg/dl (control nest) and 242.3 mg/dl (treated nests). The nestlings from treated nests had on average 31.4 mg/dl lower glucose concentration than the nestlings in control nests in both parkland and forest study areas (Table 1, Fig. 2). The mean glucose concentration also varied between both study areas and was on average 41 mg/dl higher in the parkland study area than in the forest study area in both treated and control nestlings (Table 1, Fig. 2).

The mean numbers of nestlings did not differ between treated and control nests in the urban parkland study area (8.1 ± 1.6 SD vs. 7.5 ± 1.5 nestlings/nest) (Student’s *t*-test for independence samples, *t* = 0.92, df = 21, *P* = 0.37) and in the forest study area (9.0 ± 1.4 vs. 9.0 ± 1.6 nestlings/nest) (Student’s *t*-test for independence samples, *t*= 0.07, df = 40, *P* = 0.95).

## Discussion

We found that nestlings of great tits in artificial, parasite–pathogen free nests improved their health status in comparison with control nestlings in both study areas. We confirmed that both body condition indicators, the level of haemoglobin and the level of glucose, displayed a similar (but inverse) patterns. The mean haemoglobin and glucose concentrations (treated and control) also differed between the study areas, with a higher haemoglobin level and a lower glucose concentration in the forest study area, suggesting that nestlings from the forest are characterized by better physiological condition.

Previous studies have shown that the ectoparasites may affect tit nestling health status and survival. In great and blue tits nests and nestlings, various parasites have been identified, i.e. as *Protocalliphora* (Hurtrez-Boussès *et al.*, 1997, 1998), black flies *Simuliidae* and biting midges *Culicoides sp.* (*Tomás et al.*, *2008*; Krams *et al.*, 2013), hen fleas *Ceratophyllus gallinae* (Heeb *et al.*, 1996), ticks *Ixodes* sp. (Heylen and Mattyhysen, 2011; Heylen *et al.*, 2013) or protists that belong to the family Haemosporidia, e.g. *Plasmodium*, *Haemoproteus* (transmitted by bloodsucking insects, causing infection known as avian malaria) (Krams *et al.*, 2013; Podmokła *et al.*, 2014). In the light of the life-history theory, the absence or reduction of parasite burden should be beneficial in terms of the ability to invest more energy in needs other than the reduction of the direct disadvantageous effects of parasitism, the resources used by the parasites themselves and the energy used to mount the immune response (López-Rull and Macías Garcia, 2015). Some studies show no effect of reduced parasite loads on morphometric body condition indices (Słomczyński *et al.*, 2006; *Tomás et al.*, *2008*). This suggests that physiological indicators of condition may be more sensitive to changes caused by parasites and blood characteristics seem to be preferable indicators of health status dynamics of a bird. Other studies that use haematological parameters of nestlings and adults to access to body condition indicate that birds from nests with reduced parasite loads improved their health status. Some authors use total antioxidant capacity in plasma as a physiological index of condition and stress in this context. Other possibilities are total levels of glutathione (tGSH) in red blood cells (López-Arrabé *et al.*, 2015), haemoglobin concentration (Słomczyński *et al.*, 2006; Krams *et al.*, 2013), H/L ratio (Krams *et al.*, 2013), immunoglobulin level (Tomás *et al.*, 2007), but we are not aware of using the level of glucose as a physiological index of condition in the context of experimental manipulation of parasite loads in tits.

Krams *et al.* (2013) prevented parasite vectors from biting and infecting great tit nestlings by using insect repellent inside nest boxes. They showed experimentally that in the absence of arthropod parasites the nestlings had higher concentrations of haemoglobin (nestling in nests with repellent had an average about 17 g/l higher haemoglobin level, then nestlings in nests without repellent), suggesting increased health status. Lower difference in haemoglobin concentration between experimental and control groups in our study may be related to the fact that repellent was more effective in getting rid of parasite arthropods than the use of artificial nests (perhaps in the case of nest replacement, some of invertebrates could survive in the cracks or other parts of the nest box). The second reason could be large natural variation of the mean level of haemoglobin in the blood of nestlings between years depending on food availability and weather conditions, as showed by Kaliński *et al.* (2015a)*and*Glądalski *et al.* (2016b).

Słomczyński *et al.* (2006) replaced blue tit nests with clean artificial nests (twice during the nestling stage). This treatment caused an increase of about 9 g/l in haemoglobin concentration of nestlings in comparison with control nestlings. That study was also conducted in two study areas: an urban parkland and a rich deciduous forest. The authors, in contrast to our present results, did not find a difference in haemoglobin concentrations between both study areas. Our long-term studies on tits show that there are inter-habitat differences in mean haemoglobin concentration of nestlings between the parkland and forest areas (for great tits Kaliński *et al.*, 2015a and blue tits Glądalski *et al.*, 2016b). Glądalski *et al.* (2016b) show that in the period 2003–13 only in three years (2004, 2009 and 2013), the inter-habitat variation in the level of haemoglobin did not differ between the urban parkland study area and the forest study area in blue tits (and 2004 was the year of conducting the experiment, later analysed in Słomczyński *et al.*, 2006), in all the other years the haemoglobin concentration was higher in the forest study area. Haemoglobin concentration in the blood of great tits is also higher in the forest study area then the parkland study area (Kaliński *et al.*, 2015a; in the period 2003–13 only in 2010 there was no difference). Those differences between both study sites (in the haemoglobin and glucose levels) may reflect differences in trophic conditions. Both study areas considerably differ in caterpillar productivity, with the forest site being characterized by 2–5 times higher abundance of caterpillars than the parkland site (Marciniak *et al.*, 2007; Glądalski *et al.*, 2016b). The parkland area is also more fragmented, its structure is less compact and the lights surrounding gardens (street and house lights) may lure insects out (Marciniak *et al.* 2007).

Haemoglobin concentration and glucose concentration in the present experimental study displayed similar (but inverse) patterns. Negative correlation between those two condition indicators in birds has been recently shown by Minias (2014) for adult whiskered terns *Chlidonias hybrida*, by Glądalski *et al.* (2015) for pied flycatcher *Ficedula hypoleuca* nestlings and Kaliński *et al.* (2016) for adult great and blue tits. As expected, our experimental treatment caused a decrease in glucose concentration in nestlings in comparison with control nestlings. We have no knowledge of any other experiments related to the response to experimental reduction in nest parasites in tits and the level of glucose. Therefore, our results suggests that the level of glucose in the blood of tit nestlings is a reliable index of body condition, capable of capturing subtle physiological effects of host-parasite relationships.

Our study confirms that the level of haemoglobin and especially the level of glucose may be treated as a reliable indicator of environmental characteristics in great tits. Some studies have shown that nest boxes harbour significantly higher loads of ectoparasites than natural nests in tree cavities. Probably the issue of high ectoparasite loads in nest boxes may be associated with the nest box itself rather than with an individual species (Devaynes *et al.*, 2018). Therefore, the next step would be to investigate the blood based index of physiological condition of nestlings in response to experimental reduction in natural holes nest micro- and macro-parasites.
